# Purification and Characterization of Polyhydroxyalkanoate Synthase from Extremely Halophilic Archaeon *Haloferax mediterranei*: Key Enzyme of Biodegradable Plastic Synthesis

**DOI:** 10.3390/bioengineering12091003

**Published:** 2025-09-22

**Authors:** Diya Alsafadi, Yomen Ghalawinji, Fawwaz I. Khalili

**Affiliations:** 1 Biosynthesis and Biocatalysis Research Unit, Research for Industry Center, Royal Scientific Society, Amman 11942, Jordan; 2 Department of Chemistry, The University of Jordan, Amman 11942, Jordan; yomenghalawingi@gmail.com (Y.G.); fkhalili@ju.edu.jo (F.I.K.)

**Keywords:** *Haloferax mediterranei*, PHA synthase, haloarchaea, enzyme activity, polyhydroxyalkanoates synthesis, extremophile, halophiles

## Abstract

The biosynthesis of polyhydroxyalkanoate (PHA) biopolymer is highly dependent on the activity of a key enzyme, PHA synthase (PhaC). The halophilic archaeon *Haloferax mediterranei* can accumulate large amounts of PHAs from different carbon sources under non-sterilized conditions. In this study, a PhaC enzyme from *H. mediterranei* was produced and subsequently partially purified by ion exchange chromatography. The protein was visualized by SDS-PAGE, with a subunit molecular mass of 56.4 kDa. The purified enzyme converts hydroxybutyryl CoA molecules into PHA, being optimally active at pH 10.0 and pH 8.0. The PhaC was thermoactive in the range of 30 °C to 70 °C, with maximum activity registered at 50 °C. The enzyme was confirmed to be haloalkaliphilic (active at pH > 7.0 and high salt concentration) and exhibit a degree of stability at 25 °C for 24 h.

## 1. Introduction

Plastic pollution has become a major global concern, driven by the widespread use of petroleum-based traditional plastic materials (most of them are non-recyclable) and the accumulation of microplastics and nanoplastics in aquatic, terrestrial, and atmospheric environments [1,2]. To address the plastics problem, there is growing interest in sustainable alternatives, such as polyhydroxyalkanoate (PHA) biopolymers, which exhibit plastic-like properties and are derived from microbial biomass [3]. PHAs are a broad class of polyesters accumulated in the cytoplasm of prokaryotes from the domains Bacteria and Archaea as a source of carbon and energy under unfavorable conditions (excess carbon and depletion of essential nutrients) [4,5].

A wide variety of Bacteria and Archaea have been reported to produce PHAs, occupying diverse ecosystems including the ocean, estuaries, soil, salt rocks in the mountains, and plant surfaces [4]. Indeed, haloarchaea from the Archaea domain present a novel and more economical opportunity for PHA production compared to bacterial systems. Haloarchaeal in the genera Haloferax, Halobacterium, Haloarcula, and Haloquadratum thrive optimally under high salinity conditions, which eliminates the need for sterilization during cultivation [4]. Additionally, these microorganisms offer the advantage of easy cell lysis using distilled water, which facilitates PHA granule recovery and reduces downstream processing costs [6].

Among the halophilic archaeal species, *Haloferax mediterranei* has been identified as a promising candidate for the industrial production of PHA from low-cost waste substrates [7]. Particularly, *H. mediterranei* was able to synthesize a pioneer polymer, poly(3-hydroxybutyrate-co-3-hydroxyvalerate) (PHBV), which had a wide range of applications in the fields of biomedical research, medical devices, tissue engineering, and smart food packing [8]. The PHBV synthesis was carried out through multiple pathways inside *H. mediterranei* cells without requiring expensive precursors, such as propionate or valerate.

Extensive research conducted over the past several decades has yielded comprehensive insights into the synthetic pathways of PHA and the enzymes involved in their biosynthesis and degradation process [9]. To date, an increasing number of enzymes have been identified as playing roles in the synthesis and regulation of PHA. Among these enzymes, polyhydroxyalkanoate synthase (encoded by *phaC*) is the main enzyme involved in PHA biosynthesis and is responsible for catalyzing the polymerization of 3-hydroxyacyl-coenzyme A (CoA) into PHA monomers [9]. Due to the strict stereospecificity of the PhaC enzyme, all the monomer units in the PHA backbone are produced in specific stereochemistry (R configuration) [10]. PHA synthases are generally divided into four classes according to substrate preference and subunit composition. Class I PHA synthases (PhaC) are composed of a single type of polypeptide (65 kDa), which accepts short-chain-length PHA monomers (scl-contain 3–5 carbon atoms) such as 3-hydroxybutyryl-CoA and 3-hydroxyvaleryl-CoA [11]. The PHA synthase of class I is represented by *Ralstonia eutropha,* the most studied host for this enzyme [12]. Enzymes from class II PHA synthases (PhaC1 or PhaC2) also consist of only one single type of polypeptide chain (~63 kDa); however, these enzymes catalyze the polymerization of medium-chain-length (mcl-) PHA monomers (6–14 carbon atoms). The substrates for class II PHA synthases primarily come from intermediates of fatty acid β-oxidation and de novo fatty acid biosynthesis, when fatty acids or unrelated carbon sources like carbohydrates are provided [13]. Class III synthases are heterodimers formed from PhaE and PhaC subunits with a molecular weight of around 40 kDa for each unit [12]. PHA synthases from the bacterial domain, such as *Allochromatium vinosum,* are representatives of Class III PHA synthases; however, this class was identified and characterized in Haloarcula such as *Haloarcula marismortui* [14]. Class IV PHA synthases include enzymes containing two subunits; one is PhaC, and the other is PhaE (40 kDa) or PhaR (22 kDa). This enzyme is specific to scl-PHA polymerization and found in *Bacilli* and related species [15].

In previous studies, *phaE* and *phaC* genes encoding the PHA synthase were overexpressed in *E. coli* and *H. mediterranei* [16,17,18], however there is no data available regarding the native PHA synthase enzyme from *H. mediterranei*. It is well known that the production of PHA biopolymer has been extensively studied in native (non-engineered) *H. mediterranei* cells using multiple substrates [19]. This study presents the first report on the production, purification, and biochemical characterization of the native PHA synthase from *H. mediterranei*. Additionally, the effects of temperature, pH, and salt concentration on the catalytic activity of the enzyme were investigated.

## 2. Materials and Methods

### 2.1. Chemical Reagent and Standards

All chemical reagents, unless stated otherwise, were purchased as analytical grade. DL-3-hydroxybutyryl coenzyme A lithium salt, 5,5′-dithiobis (2-nitrobenzoic acid) and Bradford protein assay dye reagent were purchased from Sigma-Aldrich, Darmstadt, Germany. A broad range protein marker, P7712S, (22–245 kDa) was purchased from New England Biolabs, Ipswich, MA, USA.

### 2.2. Microorganisms and Growth Conditions

*Haloferax mediterranei* DSM 1411 was obtained from the German Collection of Microorganisms and Cell Cultures (DSMZ), Braunschweig, Germany. The strain was maintained in nutrient-rich AS-168 medium [20] and 20% glycerol at −80 °C. The stored *H. mediterranei* cell was grown on *Hv*-YPC agar medium at 37 °C as described previously [21]. For liquid culture, *H. mediterranei* was cultivated in 300 mL highly saline medium containing (per liter) 150.0 g NaCl, 13.0 g MgCl_2_∙6H_2_O, 4.0 g KCl, 0.69 g CaCl_2_∙2H_2_O, 63 mg NH_4_Fe(III) citrate, 20.0 g MgSO_4_∙7H_2_O, 0.25 g NaHCO_3_, 0.5 g KBr, 6.25 g yeast extract (Oxoid, Manchester, UK) and 10.0 g glucose (Oxoid, Manchester, UK). The pH was adjusted to 7.2. The culture was incubated in a shaking incubator with constant shaking (170 rpm) at 37 °C. The cell growth was monitored by measuring the optical density at 520 nm (OD_520_ nm) using a Biochrom Libra S50 UV–visible spectrophotometer, Cambridge, UK. The cells were cultured for 4 days to OD_520_ nm values of approximately 2.0.

### 2.3. Production and Purification of PHA Synthase

Following *H. mediterranei* growth, cells were harvested by centrifugation at 6340× *g* for 15 min, and 2.72 g of cell pellets were obtained. The resulting pellets were resuspended in 50 mM Tris–HCl buffer, pH 8, containing NaCl (1 M) and disodium EDTA (2 mM) (1 mL buffer was used to resuspend 100 mg cells). Cells were disrupted by sonication at 6 W, 4 °C until the lysate appeared transparent. The cell lysate was centrifuged at 6340× *g* for 20 min, and the supernatant was clarified by filtration (0.45 µm). The PHA synthase enzyme was purified using Fast Liquid Protein Chromatography (GE AKTA Prime Plus FPLC System w, Uppsala, Sweden. The protein signals were visualized using Primeview 5.31 Software (GEHealthcare, cat.no.28-9949-61). The supernatant resulting from *H. mediterranei* cells was loaded at a flow rate of 0.5 mL/min onto a GE Healthcare chromatography column, 700 mm length, 16 mm ID, filled with diethylethanolamine (DEAE)-Sepharose. The column was previously equilibrated with 50 mM Tris–HCl buffer, pH 8, and contained NaCl (10 mM). An isocratic elution using 50 mM Tris–HCl buffer, pH 8, containing NaCl (2 M) was applied to elute the PHA synthase protein. The purification was monitored at a wavelength of 280 nm. Fractions (1 mL) were collected and assayed for PHA synthase activity. Active fractions were pooled and analyzed by SDS-PAGE using 12% polyacrylamide gels, stained with Coomassie brilliant blue R250. Dye unbound to protein was removed by gentle shaking in destaining solution [methanol (45% (*v*/*v*)) and glacial acetic acid (10% (*v*/*v*))] until the gel background was colorless. A broad range protein marker (11–245 kDa) was used for the determination of relative molecular weight. Protein concentration was determined using the Bradford protein assay dye reagent. Linear standard and micro assay standard curves were prepared using bovine serum albumin. Active PHA synthase fractions were pooled based on purity and specific activity and then stored at −20 °C.

### 2.4. Determination of PHA Synthase Activity

PHA synthase activity was determined based on the absorbance change upon the release of co-enzyme A from the substrate DL-3-hydroxybutyryl-CoA at 412 nm according to the method of Valentin and Steinbu [22]. The assay mixtures (1000 μL) contained 100 μL of enzyme sample, 10 μL of (10 mM) 5, 5-dithio-bis (2-nitrobenzoic acid) (DTNB), 2 μL of (12 mM) DL-3-hydroxybutyryl coenzyme, and 888 μL of buffer (50 mM Tris-HCl pH 8; 10 mM NaCl). The enzymatic reaction was started by the addition of DL-3-hydroxybutyryl-CoA at 25 °C. The changes in the optical density of the thiobenzoate anion (TNB−) resulting from the reaction of CoA substrate and DNTB were measured at 412 nm for 1 min. One unit of activity was defined as the amount of enzyme to release 1 nmole of TNB^-^ per min.

### 2.5. Characterization of PHA Synthase

The optimum pH for PHA synthase activity was determined by performing enzyme activity assays at 25 °C using the following buffers: 50 mM HCl-KCl buffer, pH 2.0; 50 mM citric acid-K_2_HPO_4_ buffer, pH 5.0; 50 mM citric acid-K_2_HPO_4_ buffer, pH 6.0; 50 mM potassium phosphate buffer, pH 7.0; 50 mM Tris-HCl, pH 8.0; 50 mM glycine-KOH buffer, for pH 9.0 and pH 10. The optimum temperature for PHA synthase was determined by performing the standard assay activity in the range of 20 °C to 80 °C using the 50 mM Tris–HCl, pH 8 buffer containing NaCl (10 mM). The effect of salt concentration was investigated using the 50 mM Tris HCl-pH 8 buffer containing 0–3 M NaCl. The stability of the purified enzyme was studied by incubating the enzyme in the presence of 2 M NaCl as a stabilizing additive at 25 °C and measuring the PHA synthase activity over time.

## 3. Results

### 3.1. Purification of PHA Synthase

**Figure 1:**
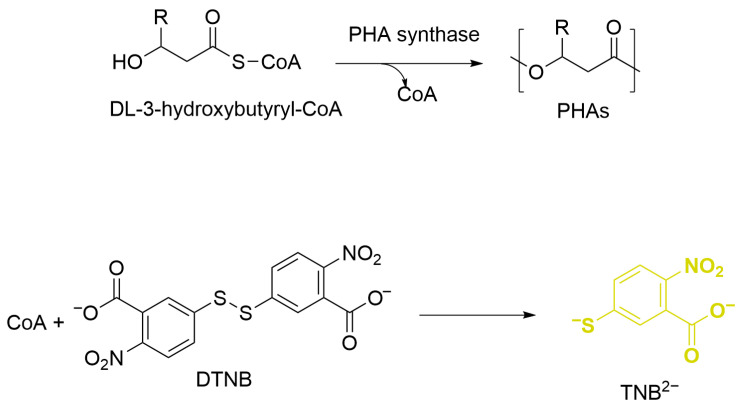
Enzymatic assay for PHA synthase using ultraviolet–visible spectroscopy at 412 nm.

PHA synthase was purified from *H. mediterranei* in one step by ion exchange chromatography, using a diethylethanolamine (DEAE)-Sepharose column. The activity of the PHA synthase was measured by monitoring the release of Coenzyme A (CoA) during the polymerization of DL-3-hydroxybutyryl-CoA substrate. This method takes advantage of the reaction between CoA and 5,5′-dithiobis-(2-nitrobenzoic acid) (DTNB), which produces a yellow colored product (TNB) with a strong absorbance at 412 nm (Figure 1).

**Figure 2:**
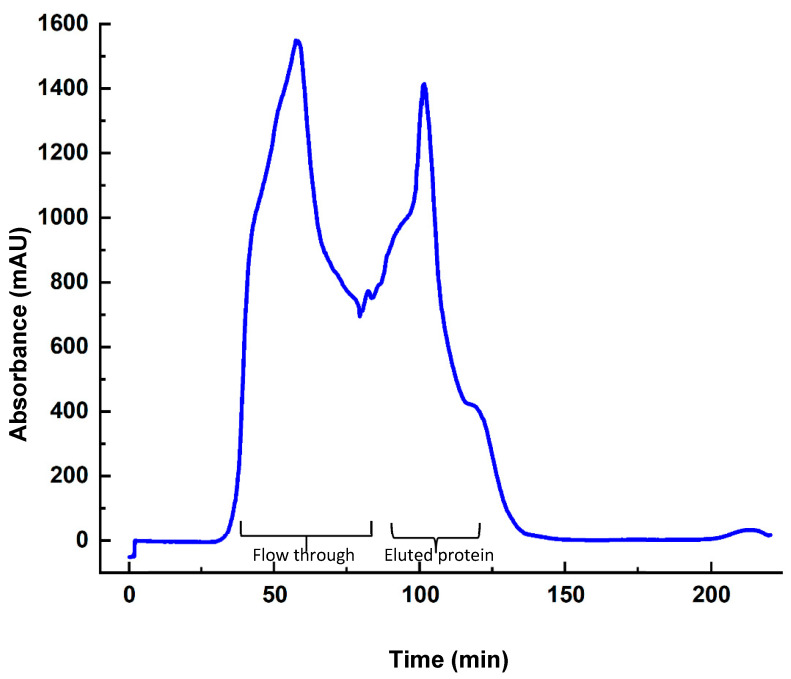
Chromatogram of PHA synthase purification by ion exchange chromatography. Peaks corresponding to the flow through and eluted protein are indicated.

Prior to purification, the specific activity of the enzyme in the crude extract was approximately 0.004 U/mg. The PHA synthase supernatant was loaded at a flow rate of 0.5 mL/min onto DEAE-Sepharose anion exchange chromatography resin. The column was previously equilibrated with buffer (50 mM Tris–HCl buffer, pH 8), and contained 10 mM NaCl. Prior to protein elution, unspecifically bound protein was removed by the passage through the column (Figure 2). The passage of protein from the column was detected spectrophotometrically at 412 nm, enabling the monitoring of the purification by means of a chromatogram. No activity was detected in the flow-through, confirming the binding of the PHA synthase to the DEAE-Sepharose resin. The enzyme was eluted by the passing of the elution buffer (50 mM Tris-HCl buffer, pH 8.0), containing 2 M NaCl through the column at a flow rate of 0.5 mL/min. Fractions (1 mL) were collected during the elution process. It was observed that the enzyme eluted at 20 mL of elution buffer (Figure 2).

**Figure 3:**
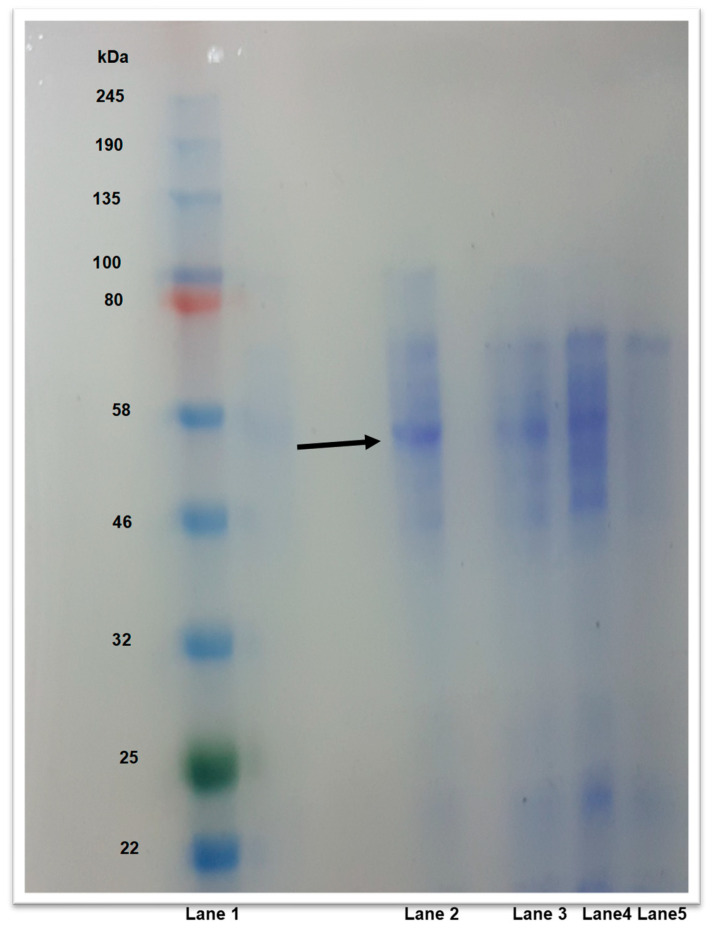
SDS-PAGE analysis of the PHA synthase from *H. mediterranei*. Lane 1: A broad range protein marker, P7712S. Lanes 2–5: active PHA synthase fractions. Molecular masses in kilodalton are indicated. An arrowhead indicates the position of the PHA synthase.

Several active fractions were collected and the most active fractions were pooled and visualized by sodium dodecyl sulfate polyacrylamide gel electrophoresis (SDS-PAGE) (Figure 3), which revealed a band approximately corresponding to the subunit molecular mass of 56.4 kDa. This molecular mass is consistent with the expected molecular mass (54.8 kDa) of the catalytic subunit (PhaC) of the PHA synthase from *H. mediterranei* [23]. Interestingly, this catalytic subunit (PhaC) was not observed in any of the purified PHA synthase fractions from the homologous overexpression of the PHA synthase of *H. mediterranei* using a native expression system [16]. PHA synthases classified as Class I and II typically have molecular masses ranging from approximately 63 to 73 kDa. In contrast, Class III synthases consist of two distinct subunits, PhaC and PhaE, each with a molecular mass of about 40 kDa [12,13]. The PHA synthase from *H. mediterranei* had an apparent molecular mass of 54.8 kDa and may represent a novel class of PHA synthases.

Following the purification of the PHA synthase, the specific activity was found to be 0.01 U/mg, indicating a purification factor of 2.5. Pantazaki et al., reported the specific activity of the PHA synthase in the initial extract from the thermophilic bacterium *Thermus thermophilus* at a value of 0.48 U/mg [24]. According to Reddy et al., *P. palleronii* exhibited higher PHA synthase activity (0.142 U/mL) compared to *P. pseudoflava* (0.054 U/mL). Additionally, the protein concentration obtained from *P. palleronii* (0.68 mg/mL) was higher than in *P. pseudoflava* (0.57 mg/mL) [25]. Bhubalanet al. reported a highly active PHA synthase from *Chromobacterium* sp. USM2 with a specific activity value of 238 ± 98 U/mg [26]. The activity of this natural synthase was found to be higher than that of some of the engineered mutant synthases, such as purified PhaC from *C. necator* [26]. The variation in catalytic activity of PHA synthase could be attributed to the enzyme source; for example, the PHA synthases from thermophilic Bacteria and Archaea showed higher thermostability and catalytic activity than their mesophilic counterparts [27]. External conditions, such as light, could affect PHA synthases’ activity. The enzyme activity of poly-β-hydroxybutyrate (PHB) synthesis was detected exclusively in the membrane fractions of nitrogen-deprived cells of the *Synechococcus* sp. strain (MA19) under light conditions, and was not observed in the dark. The shift in activity was unaffected by chloramphenicol, suggesting that the activation occurs posttranslationally [28]. The activity of some PHA synthases is associated with their form; for example, Yuan et al. reported the purification of inactive PHA synthase in a soluble form from *R. eutropha*, while the granule-bound form retained the enzymatic function [29]. Finally, PHA synthase activity could be significantly influenced by abiotic factors such as pH, temperature, and ionic strength, which affect the enzyme’s structure and catalytic efficiency. Therefore, these factors were investigated in this study.

### 3.2. Characterizationof PHA Synthase

**Figure 4:**
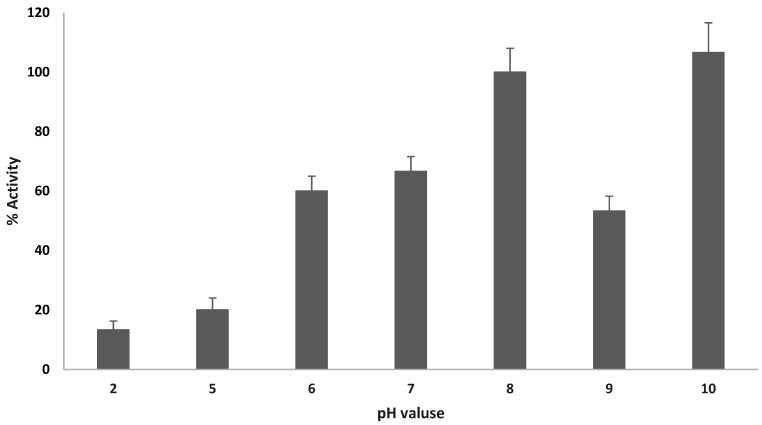
The relative activity of PHAs synthase at different pH values. 100% activity corresponded to a specific activity of 0.01 U/mg.

The purified PHA synthase from *Haloferax mediterranei* was screened to determine its optimal activity conditions. Initially, enzyme activity was assayed across a pH range of 2.0 to 10.0. In a previous study, the PHA synthase from *Thermus thermophilus* exhibited optimal activity around pH 7.3 [24], while the highest activity in the enzyme from *Arthrospira platensis* was observed in 100 mM Tris-HCl buffer at pH 8.0 [30]. In this study, the PHA synthase from *H. mediterranei* showed maximum activity at both pH 8.0 and pH 10.0 (Figure 4), indicating that it is an alkaliphilic enzyme. It should be noted that buffer composition can influence enzyme activity independently of pH. Therefore, future work will examine potential enzyme–buffer interactions in more detail.

**Figure 5:**
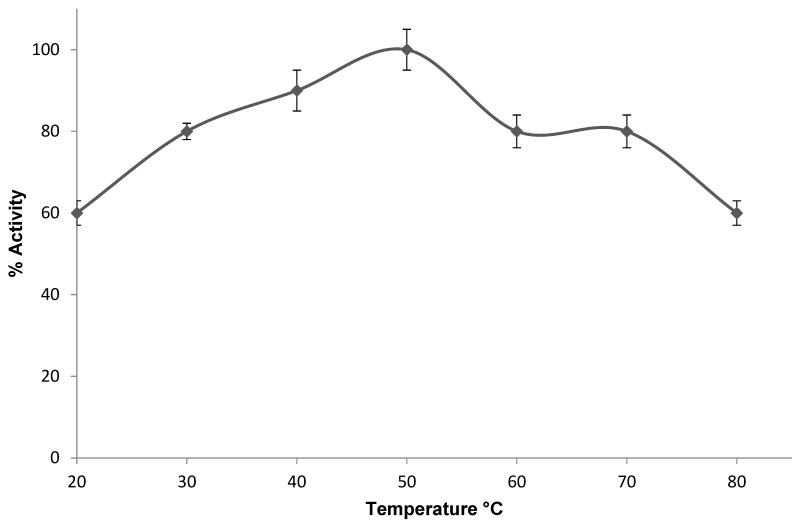
Thermoactivity of PHA synthase from *H. mediterranei*. 100% activity corresponded to a specific activity of 0.01 U/mg.

While the optimum temperatures for the activity of enzymes from halophilic archaeal are often not as high as those tolerated by thermophilic enzymes, halophilic enzymes are certainly more thermoactive than their mesophilic counterparts, thereby adding another dimension to their industrial applicability. Figure 5 shows the activity registered by the PHA synthase from *H. mediterranei* between 20 and 80 °C. The maximum activity was observed at 50 °C. Above this temperature, the enzyme’s activity decreased, and the reproducibility of results was compromised. This temperature was lower than the optimal temperature (70 °C) of the PHA synthase activity isolated from *Thermus thermophilus* [24].

**Figure 6:**
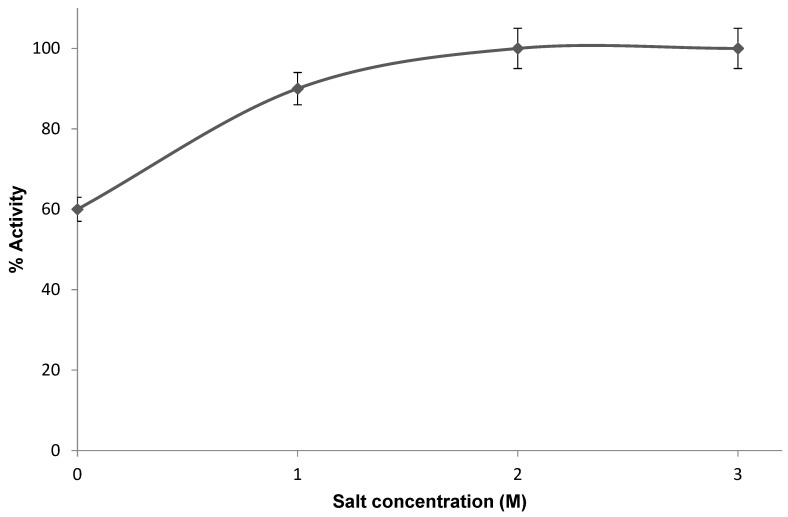
The activity of PHA synthase at different salt concentrations. 100% activity corresponded to a specific activity of 0.01 U/mg.

In a previous study, the activity of the PHA synthase from *Ralstonia eutropha* was found to decrease with increasing buffer concentration. Therefore, this enzyme was maintained in a buffer with moderate ionic strength (not exceeding ~100 mM). In this study, the PHA synthase from *H. mediterranei* showed high activity in high salt concentrations, up to 3.0 M NaCl (Figure 6). This is not surprising, since the PHA synthase from *H. mediterranei* is a halophilic protein. The high salt concentrations within the cytoplasm of halophilic cells necessitate that their proteins evolve specialized mechanisms to maintain their native conformation and remain functionally active [31]. With respect to their non-halophilic counterparts, halophilic proteins feature an excess of acidic residues over basic residues, predominantly located on the protein surface. They also have a lower lysine content, an increase in small hydrophobic residues, and a decrease in aliphatic residues in comparison to their non-halophilic counterparts [32]. The structure of the PHA synthase from *H. mediterranei* could be explored in more detail in future studies through X-ray crystallography for a better understanding of these adaptations. Furthermore, the stability of the purified enzyme over time was investigated at 25 °C. The enzyme retained approximately 20% of its original activity after 3 days.

## 4. Discussion

The production of PHA has been described in a number of prokaryotic microbes, including various extremophiles [9]. Nevertheless, extremophiles represent more promising candidates for commercial PHA production, as their ability to reduce contamination risks and enable phase-shift production can significantly lower costs. This was explained by the “Next-Generation Industrial Biotechnology” (NGIB) concept. In fact, both halophiles and thermophiles appear to be the most promising extremophiles for PHA production. These extremophilic microorganisms are not only a promising biotechnological chassis for direct PHA production, but also valuable sources of genes and enzymes for both in vivo and in vitro PHA synthesis [4]. These thermophiles have been at the forefront in terms of their biotechnological applications in this field, and the most investigated extremophilic PHA synthesis enzymes with superior stability, activity [27], and substrate specificity [24] are those from thermophilic microorganisms.

In this study, we purified and characterized the native PHA synthase enzyme from halophilic archaeal *H. mediterranei* for the first time. The enzyme was partially purified successfully in one step by ion exchange chromatography. The purified fractions showed higher activity than the crude extract. It is important to note that higher PHA synthase activity is generally associated with increased PHA biopolymer accumulation. The enzyme was confirmed to be haloalkaliphilic (active at pH > 7.0 and at high salt concentration) and thermoactive, being optimally active at pH 8 and pH 10.0 in the presence of 2–3 M NaCl at 50 °C. Thermoactivity is a critical property of enzymes used in the in vitro production of PHA, as denaturation can occur during the reaction process. Moreover, higher reaction temperatures enhance reaction rates, making thermostable and thermoactive enzymes essential for achieving high product yields [27]. The thermoactivity profile of PHA synthase aligned with the optimal temperature range (37–49 °C) for both the growth and PHA production of *H. mediterranei* [33].

In conclusion, the ability of *H. mediterranei* to produce PHA efficiently is strongly related to the unique properties of its native PHA synthase enzyme. This enzyme’s activity under high-salt, alkaline, and moderately high-temperature conditions reflects the organism’s adaptation to extreme environments and directly supports its capacity for robust PHA biosynthesis. The successful purification and characterization of this haloalkaliphilic and thermoactive PHA synthase further confirm the biotechnological potential of *H. mediterranei* as a high-performance chassis for sustainable and large-scale PHA production.

## Data Availability

Data is contained within the article.
